# High Ambient Temperature Aggravates Experimental Autoimmune Uveitis Symptoms

**DOI:** 10.3389/fcell.2021.629306

**Published:** 2021-03-25

**Authors:** Su Pan, Handan Tan, Rui Chang, Qingfeng Wang, Ying Zhu, Lin Chen, Hongxi Li, Guannan Su, Chunjiang Zhou, Qingfeng Cao, Aize Kijlstra, Peizeng Yang

**Affiliations:** ^1^The First Affiliated Hospital of Chongqing Medical University, Chongqing Key Lab of Ophthalmology, Chongqing Eye Institute, Chongqing Branch of National Clinical Research Center for Ocular Diseases, Chongqing, China; ^2^University Eye Clinic Maastricht, Maastricht, Netherlands

**Keywords:** experimental autoimmune uveitis, inflammation, fumaric acid, succinic acid, ambient temperature

## Abstract

Whether ambient temperature influences immune responses leading to uveitis is unknown. We thus tested whether ambient temperature affects the symptoms of experimental autoimmune uveitis (EAU) in mice and investigated possible mechanisms. C57BL/6 mice were kept at a normal (22°C) or high temperature (30°C) housing conditions for 2 weeks and were then immunized with human interphotoreceptor retinoid-binding protein (IRBP651–670) peptide to induce EAU. Histological changes were monitored to evaluate the severity of uveitis. Frequency of Th1 cells and Th17 cells was measured by flow cytometry (FCM). The expression of IFN-γ and IL-17A mRNA was measured by real-time qPCR. The generation of neutrophil extracellular traps (NETs) was quantified by enzyme-linked immunosorbent assay (ELISA). Differential metabolites in the plasma of the mice kept in the aforementioned two ambient temperatures were measured via ultra-high-performance liquid chromatography triple quadrupole mass spectrometry quadrupole time of flight mass spectrometry (UHPLC-QQQ/MS). The differential metabolites identified were used to evaluate their effects on differentiation of Th1 and Th17 cells and generation of NETs in vitro. The results showed that EAU mice kept at high temperature experienced a more severe histopathological manifestation of uveitis than mice kept at a normal temperature. A significantly increased frequency of Th1 and Th17 cells in association with an upregulated expression of IFN-γ and IL-17A mRNA was observed in the splenic lymphocytes and retinas of EAU mice in high temperature. The expression of NETs as evidenced by myeloperoxidase (MPO) and neutrophil elastase (NE), was significantly elevated in serum and supernatants of neutrophils from EAU mice kept at high temperature compared to the normal temperature group. The metabolites in the plasma from EAU mice, fumaric acid and succinic acid, were markedly increased in the high temperature group and could induce the generation of NETs via the NADPH oxidase-dependent pathway, but did not influence the frequency of Th1 and Th17 cells. Our findings suggest that an increased ambient temperature is a risk factor for the development of uveitis. This is associated with the induction of Th1 and Th17 cells as well as the generation of NETs which could be mediated by the NADPH oxidase-dependent pathway.

## Introduction

Uveitis is an intraocular inflammation which can be caused by infectious and non-infectious mechanisms. Non-infectious uveitis is thought to be caused by an autoimmune or auto-inflammatory response and is often difficult to diagnose and if not appropriately managed may lead to irreversible blindness, causing a heavy burden to patients and their families (Yang et al., 2005). Th1 and Th17 cells and activated neutrophils play an important role in the pathogenesis of uveitis (Balkarli et al., 2016; Safi et al., 2018; Chang et al., 2020).

Both genetic as well as environmental factors can influence the risk of developing an autoimmune disease such as uveitis (Zhou et al., 2014; Tan et al., 2020). Known environmental factors in uveitis include cigarette smoking and vitamin D status (Yuen et al., 2015; Chiu et al., 2020). One of the environmental factors that has not been looked at in detail is the role of climate. Climate change is currently recognized as a serious, worldwide public health concern and some diseases have been shown to be associated with environmental temperature and humidity (Burke et al., 2018; Gao et al., 2019). For example, relatively cool ambient housing temperature could induce suppression of the antitumor immune response and promotes tumor growth and higher temperature may accelerate atherosclerosis (Kokolus et al., 2013; Tian Xiao et al., 2016). To date, only few studies have addressed whether ambient temperature can influence the occurrence of autoimmune disease. We recently reported on the role of climate change in mainland China and showed that a gradual increase in temperature is associated with an increased incidence of uveitis and found that a 1°C increase in monthly temperature was associated with a rise in approximately two uveitis reports per 1,000 individuals (Tan et al., 2020). How temperature affects the development of uveitis is not clear and was therefore the subject of the study presented here, where we used an experimental autoimmune uveitis (EAU) model in mice (Agarwal et al., 2012). We studied the effect of temperature on the autoimmune response to a retinal peptide and focused on the role of T cells and neutrophils as well as the differential expression of metabolites. Our results show that housing mice at a higher temperature is associated with a more severe uveitis. Analysis of the involved mechanisms show that an increase of the ambient temperature is associated with a higher Th1 and Th17 cell response and an increased generation of neutrophils extracellular traps (NETs). Analysis of metabolites suggests important roles for fumaric acid and succinic acid.

## Materials and Methods

### Housing Mice at Different Temperatures

Female, 4-week old C57BL/6J mice were obtained from the Chongqing Medicine University, Department of Laboratory Animal Resources. The mice were housed 6 to a cage in manual climatic boxes (Wanfeng Instrument, Jiangsu, China) maintained at a normal (22°C) or high temperature (30°C) environment. Humidity was controlled at 40 to 50%. Lights were adjusted to 12 h of daylight and 12 h of darkness. Mice were kept under these conditions for 14 days prior to the induction of uveitis (Kokolus et al., 2013).

### Experimental Autoimmune Uveoretinitis Model

The mice above were immunized with 350 μg human IRBP peptide (IRBP651–670, LAQGAYRTAVDLESLASQLT) in Complete Freund’s adjuvant (CFA) (Mattapallil et al., 2015). The peptide was purchased from Sangon Biotech (Sangon Biotech, Shanghai, China). Simultaneously, the mice were injected intraperitoneally with pertussis toxin (1 μg, Sigma-Aldrich, St. Louis, MO, United States) (Van den Broeck et al., 2006; Agarwal et al., 2012). After immunization, the mice were continuously housed in their previous environments, normal or high temperature environments, respectively. In some experiments, mice were housed under normal temperature conditions and transferred to a high temperature environment after immunization with IRBP, and this group was called the heat stress group. The normal temperature group was regarded as the control group in this study. Experimental Autoimmune Uveoretinitis (EAU) mice were euthanized, and spleens, serum and retina were collected on day 14 following immunization. Earlier experiments showed that this was the peak of inflammation in EAU (Mattapallil et al., 2015; Huang et al., 2018). In total we used 64 mice and actual numbers used in the various experiments are shown in the figure legends.

The protocols were approved by the Animal Care and Use Committee of The First Affiliated Hospital of Chongqing Medical University. Efforts were made to minimize animal discomfort.

### Histological Scoring of EAU

Hematoxylin and eosin (H&E) staining. Eyeballs from EAU mice (day 14 after IRBP immunization) were dissected and fixed with paraformaldehyde. Eyes were then washed, dehydrated and embedded in paraffin wax. Serial 6 μm sections were stained with H&E and scored according to Caspi’s criteria (Agarwal et al., 2012). H&E images were made using an inverted fluorescence microscope (LEICA, DMIL4000, Germany). The severity of EAU was scored on a scale of 0 (no disease) to 4 (severe disease) by an independent ophthalmologist in a masked fashion.

### Isolation of Serum, Neutrophils and CD4^+^ T Cells

The mice were anesthetized with pentobarbital (10 mg/ml) and blood was collected from the retro-orbital plexus on day 14 after IRBP immunization. Blood was clotted at room temperature for 2 h and centrifuged at 3000 rpm for 10 min. Serum was separated and stored at −80°C and used later for enzyme-linked immunosorbent assay (ELISA).

To isolate splenic neutrophils and CD4^+^ T cells, anaesthetized mice were euthanized by cervical dislocation and splenic cell suspensions were obtained on day 14 after IRBP immunization. Lymphocyte separation solution (TBD, Tianjin, China) was used to isolate lymphocytes from the middle layer and neutrophils from the lower layer after removing erythrocytes with RBC lysis buffer (Absin, abs9101, Shanghai, China). CD4^+^ T cells were isolated from splenic lymphocytes with CD4 microbeads (Miltenyi Biotec, Bergisch Gladbach, Germany).

### Neutrophil Stimulation

Neutrophils were plated in 24-well plates at a density of 1 × 10^6^ cells/well in 1 ml RPMI 1640 complete medium containing fumaric acid (173 μM), succinic acid (500 μM) or PMA (200 ng/ml). Concentrations were based on previous studies, and cells were incubated at 37°C with 5% CO2 for 4 h (Rubic et al., 2008; Wang et al., 2017; Hoffmann et al., 2018). In other experiments, neutrophils were blocked with diphenyleneiodonium chloride (DPI, 5 μM. Selleck, United States), the inhibitor of NADPH oxidase, for 30 min prior to stimulation with fumaric acid or succinic acid (Dabrowska et al., 2016; Wang L. et al., 2019). The supernatants were collected for ELISA.

CD4^+^ T cells were plated at a density of 1 × 10^6^ cells/well in 24-well plates in 1 ml RPMI 1640 complete medium containing fumaric acid (173 μM) or dimethyl sulfoxide (DMSO,ST038, BYT, Shanghai, China) as a vehicle control. Cells were stimulated with anti-CD3/CD28 (BioLegend, 100201 and 102101, San Diego, United States) and incubated for 72 h (Huang et al., 2020). CD4^+^ T cells were collected for flow cytometry (FCM).

### ELISA

To quantify NET release, the concentrations of MPO and NE in the serum or neutrophil supernatants were assayed with an ELISA kit (R&D Systems, DY3667 and DY4517, MN, United States) according to the manufacturer’s instructions.

### Immunofluorescence Detection of NET Formation

The eyeballs from EAU mice were dissected and paraffin-embedded for further experiments. After sections were dewaxed and hydrated, a primary antibody against MPO (Abcam, ab9535, Cambridge, United Kingdom) was used to stain the NETs and DAPI (Absin, abs9235) was used to stain the nuclei overnight at 4°C avoiding light. Sections were then incubated with secondary antibodies (Alexa Fluor 555, ab150078) for 2 h at room temperature. The location of NETs in retinal tissue was detected using immunofluorescence microscopy (NIKON Eclipse ci, Japan).

### FCM

For IL-17A and IFN-γ staining, splenic lymphocytes were stimulated with ionomycin, PMA and brefeldin A (Biolegend, 423304) for 6 h, then washed, fixed and permeabilized. Fluorescent anti-mouse CD4-FITC (11-0041-82), anti-mouse IL-17A-PE (12-7177-81) and anti-mouse IFN-γ-PE-cy7 (25-7311-82) were purchased from eBiosciences (California, United States). Cells were stained with antibodies at 4°C for 30 or 60 min. The following markers were used to identify different immune cell subsets: Th1: CD4^+^IFN-γ^+^, Th17: CD4^+^IL-17A^+^. Stained cells were analyzed with CytExpert cytometry analysis software (Beckman Coulter, United States).

### RT-qPCR

RNA was extracted from splenic lymphocytes (1 × 10^7^ cells) or retinal tissue with the TRIzol reagent (Roche, 11667165001, Mannheim, Germany). The PrimeScript RT reagent Kit (MedChemExpress, HY-K0511, United States) was used to generate cDNA. Real-time qPCR was performed with the ABI Prism 7500 system (Applied Biosystems, CA, United States) by using the iTaq Universal SYBR Green Supermix (MedChemExpress, HY-K0522, United States) (Chang et al., 2018). PCR primers employed were as follows: IFN-γ: 5′-CTGCTGATGGGAGGAGATGT-3′(forward) and 5′-T TTGTCATTCGGGTGTAGTCA-3′(reverse); IL-17A: 5′-GGACT CTCCACCGCAATGA-3′(forward) and 5′-TCAGGCTCCCT CTTCAGGAC-3′(reverse) (Meng et al., 2017). PCR primers and mouse GAPDH endogenous reference gene primers were designed and purchased from Sangon Biotech (Sangon, Shanghai, China). Relative mRNA expression was calculated utilizing the 2^–ΔΔCt^ method.

### Liquid Chromatography-Mass Spectrometry (LC-MS)

The metabolomics analysis of plasma from mice kept at high or normal temperature was tested by UHPLC-QQQ/MS and was performed by the BIOTREE company (Shanghai, China). Each group had 10 samples. In brief, mice were anesthetized with pentobarbital and blood was collected from the retro-orbital plexus in heparin sodium anticoagulant tubes and centrifuged at 3000 rpm for 10 min. Plasma was separated and stored at −80°C for future LC/MS analysis. The quality control sample, was a pooled preparation, made by mixing an equal aliquot of the supernatants from all samples. The data were analyzed by the SIMCA15.0.2 software package (Sartorius Stedim Data Analytics AB, Umea, Sweden). Metabolite composition was analyzed by calculating the variable importance in the projection (VIP) of the first principal component in orthogonal partial least squares discrimination analysis (OPLS-DA). A metabolite was considered to be differentially expressed when VIP > 1 and *P* < 0.05 (Student *t*-test).

### Statistical Analysis

The results are shown as mean ± standard deviation (SD). Results that did not assume a Gaussian distribution are shown as median. One-way ANOVA and Kruskal–Wallis tests were used to perform multiple group comparisons. The unpaired *t*-test and Mann Whitney test were used to analyze two independent groups. Statistical analyses were performed with GraphPad Prism 7.0 software (GraphPad Software, Inc., CA, United States). Significance at each comparison point was indicated as: ^∗^*p* < 0.05, ^∗∗^*p* < 0.01, and ^∗∗∗^*p* < 0.001.

## Results

### High Temperature Worsens EAU Manifestation

**FIGURE 1:**
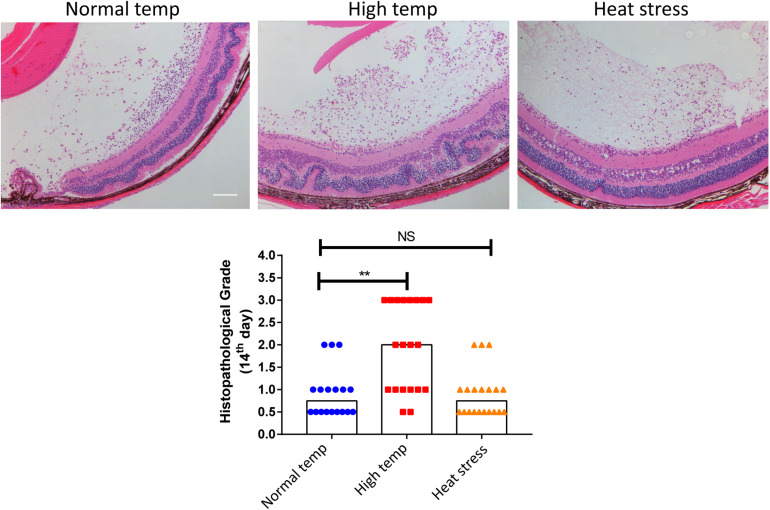
A high temperature environment aggravates ocular inflammation in EAU mice. Left, representative hematoxylin and eosin images of eye sections from EAU mice (day 14 after IRBP immunization) kept at normal temperature, high temperature or a so-called heat stress environment. Scale bar, 10 μm. Quantification of the histopathological score. Representative data from 3 independent experiments (*n*_normal temp_ = 18, *n*_high temp_ = 20, *n*_heat stess_ = 20; median; **p < 0.01; NS, no statistical differences; differences were assessed by the Kruskal–Wallis test).

To investigate whether a higher housing temperature can influence the inflammatory response during EAU, we kept mice at normal (22 degrees Celsius) or high temperature conditions (30 degrees Celsius) for 14 days and then immunized them with the retinal IRBP peptide to induce EAU. Histological scores of EAU were examined by H&E staining. The high temperature EAU mice developed significant inflammation with higher histological scores as compared to animals kept at a normal temperature (Figure 1).

In a separate experiment where we tested the effect of short duration of high temperature treatment on EAU, the mice were housed under normal temperature conditions and were then transferred to a high temperature environment after immunization with IRBP (heat stress). There was no statistical difference between the normal temperature group and heat stress group (Figure 1). Therefore, the subsequent experiments were done in mice kept at either normal or high temperature housing conditions for at least 14 days before inducing EAU.

### High Temperature Is Associated With Increased Frequencies of Th1 and Th17 Cells in EAU Mice

**FIGURE 2:**
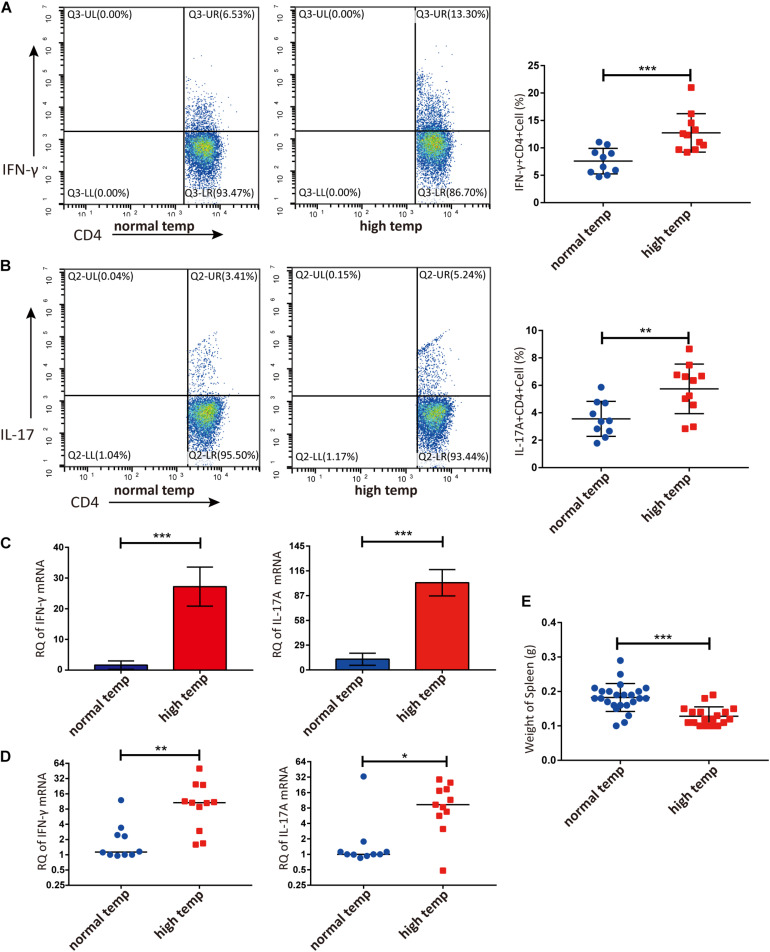
A high temperature environment changes the frequencies of Th1 and Th17 cells in the spleen and retinas of EAU mice (day 14 after IRBP immunization). **(A)** Representative flow cytometry and quantification of IFN-γ+CD4^+^ T cells in splenic lymphocytes of EAU mice (*n*_normal temp_ = 10, *n*_high temp_ = 11; mean ± SD; ***p < 0.001; Unpaired *t*-test). **(B)** Representative and quantification of IL-17A+CD4^+^ T cells in the splenic lymphocytes of EAU mice (*n*_normal temp_ = 10, *n*_high temp_ = 11; mean ± SD; **p < 0.01; Unpaired *t*-test). **(C)** The expression of IFN-γ, IL-17A mRNA were detected by real-time qPCR in splenic lymphocytes from EAU mice housed under normal or high temperature conditions (*n* = 5/group; mean ± SD; ***p < 0.001; Unpaired *t*-test). **(D)** The expression of IFN-γ and IL-17A mRNA was detected by real-time qPCR in the retina from EAU mice (*n*_normal temp_ = 10; *n*_high temp_ = 11; data were not normally distributed; median; **p < 0.01, *p < 0.05; Differences were assessed by Mann Whitney test). **(E)** Weight of spleens were compared between EAU mice under high or normal temperature housing conditions (*n*_normal temp_ = 24, *n*_high temp_ = 18; mean ± SD; ***p < 0.001; Differences were assessed by Unpaired *t*-test).

Previous studies have demonstrated that the spleen is an important immune organ and as such hosts a wide range of immunological functions (Lewis et al., 2019). We therefore focused on the effect of housing temperature conditions on the frequencies of lymphocyte subpopulations in the spleen of mice with EAU. The frequencies of Th1 and Th17 cells were significantly higher in the high temperature group when compared to the normal temperature group (Figures 2A,B). The expression of IFN-γ and IL-17A mRNA in splenic lymphocytes was also significantly increased in the high temperature group (Figure 2C). Analysis of retinal samples also showed that the expression of IFN-γ and IL-17A mRNA was significantly increased in the high temperature group (Figure 2D). During dissection, we found that the spleens of EAU mice kept at high temperatures were clearly smaller and lighter than in the normal temperature group (Figure 2E).

### High Temperature Increases the Formation of NETs

**FIGURE 3:**
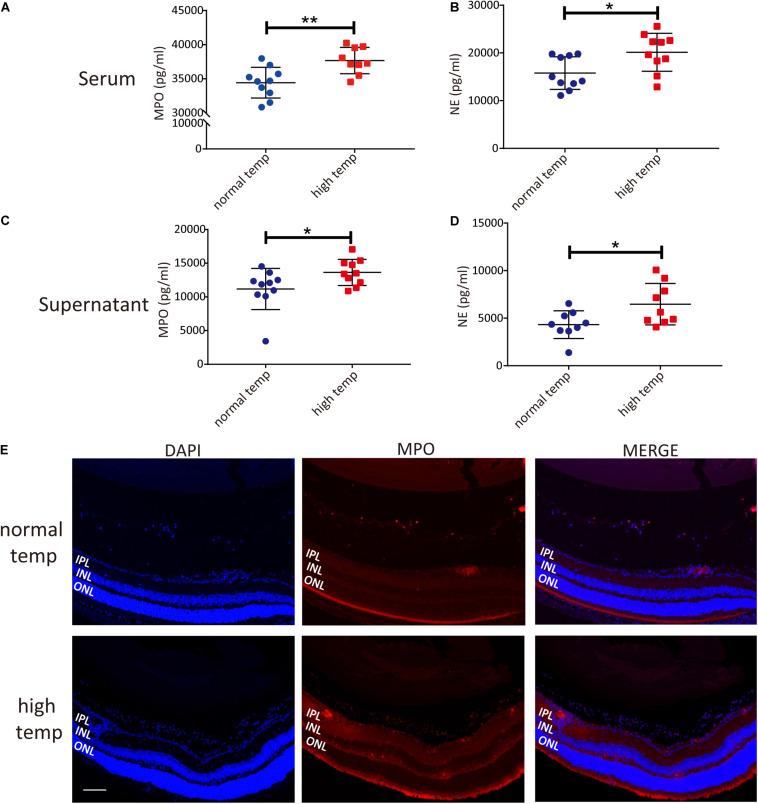
A high temperature environment induces the generation of NETs in EAU mice. **(A)** Quantification of MPO in the serum of EAU mice (*n*_normal temp_ = 10; *n*_high temp_ = 9; mean ± SD; **p < 0.01, *p < 0.05; Differences were assessed by Unpaired *t*-test). **(B)** Quantification of NE in the serum of EAU mice (*n* = 10/group; mean ± SD; **p < 0.01, *p < 0.05; Differences were assessed by Unpaired *t*-test). **(C)** Quantification of MPO in neutrophil supernatants of EAU mice (*n* = 10/group; mean ± SD; *p < 0.05; Differences were assessed by Unpaired *t*-test). **(D)** Quantification of NE in neutrophil supernatants of EAU mice (*n* = 9/group; mean ± SD; *p < 0.05; Differences were assessed by Unpaired *t*-test). **(E)** Immunofluorescence analysis of MPO to label the location of NETs in the retinas of EAU mice. Scale bar, 106 μm (IPL: inner plexiform layer, INL: inner nuclear layer, ONL: outer nuclear layer).

To investigate changes in neutrophil function following an increased housing temperature we tested MPO and NE as NET markers in serum and neutrophils from EAU animals kept at normal and high temperatures. The results showed that there was a significantly increased generation of NETs both in the serum and neutrophil supernatants from EAU mice kept under high temperature conditions (Figures 3A–D). To investigate the expression of NETs in the retinas of EAU mice, we stained the sections with antibodies against MPO. MPO immunoreactivity was more prominently detected in the high temperature group and was distributed in the vitreous, inner plexiform layer (IPL) and inner nuclear layer (INL). In the normal temperature group, a light MPO staining was observed in the vitreous and sites with neovascularization (Figure 3E). However, the staining for MPO in retinal tissues was very light and the differences were not easily quantified. Collectively, these results suggest that a high temperature environment can up-regulate the generation of NETs in EAU mice.

### High Temperature Is Associated With Higher Levels of Fumaric Acid and Succinic Acid

**FIGURE 4:**
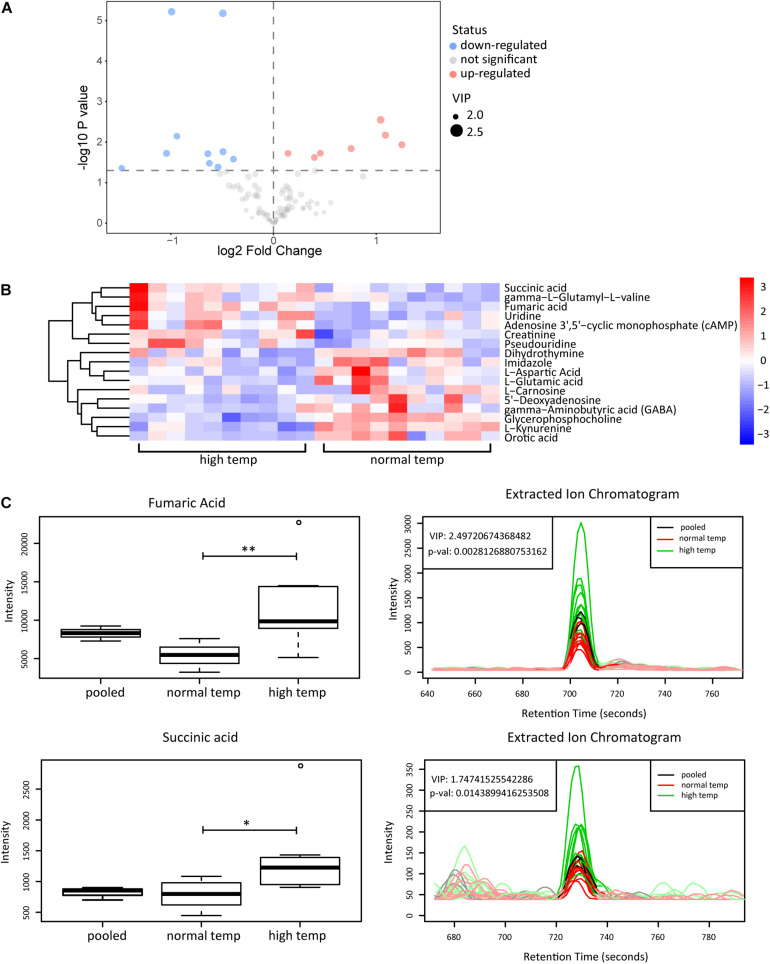
Metabolomic analyses of plasma from EAU mice housed in normal or high temperature conditions. **(A)** The upregulated (red) and downregulated (blue) metabolites between the high and normal temperature group are shown in the volcano plots. **(B)** Heatmap of the 17 significantly differential metabolites. The color is positively correlated with the intensity of change in metabolites, with red indicating up-regulation and blue indicating down-regulation. **(C)** The response of fumaric acid and succinic acid in the sample (left) and extracted ion chromatogram (right) between the high (green) and normal temperature (red) group. The relative quantitative results and extracted ion chromatogram are shown. Pooled represents the quality control sample (*n* = 10/group; **p < 0.01, *p < 0.05).

To determine the change in metabolism associated with the housing temperature conditions we analyzed plasma samples with LC-MS in two groups of 10 EAU mice (normal and high temperature). Using the OPLS-DA model, we found a significant difference in the metabolic phenotype between the two groups, suggesting that a distinct metabolic profile might exist in mice at the two different temperatures investigated (Supplementary Figure 1). Among the 141 metabolites detected in plasma, we found 17 differentially expressed metabolites as shown in volcano plots (Figure 4A). Each point in the volcanic map represents a metabolite, and the size of the scatter represents the OPLS-DA value. The larger the scatter, the greater the VIP. Higher levels of fumaric acid and succinic acid were found in the high temperature environment (Figures 4B,C). Fumaric acid and succinic acid are metabolites originating from the TCA cycle and subsequent experiments were performed to investigate whether these metabolites might affect neutrophil function.

### Fumaric Acid or Succinic Acid Promotes the Formation of NETs *in vitro*

**FIGURE 5:**
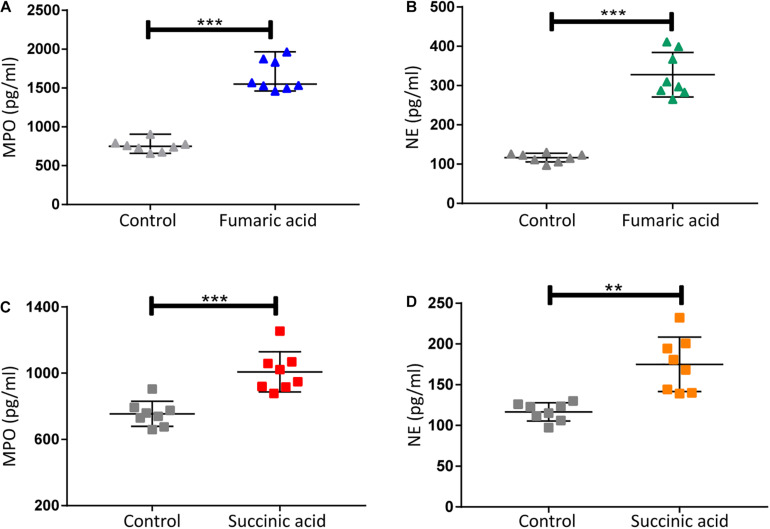
Fumaric acid or succinic acid can promote formation of NETs. **(A,B)** Quantification of MPO and NE in the supernatants of neutrophils from normal mice treated with fumaric acid or DMSO as control (*n* = 8/group; mean ± SD; ***p < 0.001; Differences were assessed by Unpaired t-test). **(C,D)** Quantification of MPO and NE in the supernatants of neutrophils from normal mice treated with succinic acid or DMSO as control (*n* = 8/group; mean ± SD; ***p < 0.001, **p < 0.01; Differences were assessed by Unpaired *t*-test).

Previous research has shown that metabolites of the TCA cycle, especially succinic acid and fumaric acid may play an important role in inflammation (Sudarshan et al., 2009; Mills and O’Neill, 2014). We therefore investigated the effect of fumaric acid or succinic acid on the generation of NETs in vitro. Splenic neutrophils from normal mice were stimulated with PMA following incubation with fumaric acid or succinic acid, but this did not affect the generation of NETs (Supplementary Figure 2). To exclude the possible influence from PMA and to test the individual effect of fumaric acid or succinic acid on the formation of NETs, we isolated neutrophils and incubated them in medium with fumaric acid or succinic acid alone, to investigate the correlation between NET formation and these two metabolites. During the 4 h of incubation under fumaric acid or succinic acid conditions, a significantly increased NET expression was observed as evidenced by the higher release of MPO and NE (Figure 5). These data indicate that fumaric acid and succinic acid can induce NET generation by neutrophils and confirm the correlation found between intermediate metabolites of the TCA cycle and NET production.

### Fumaric Acid or Succinic Acid Induces the Formation of NETs via the NADPH Oxidase-Dependent Pathway

**FIGURE 6:**
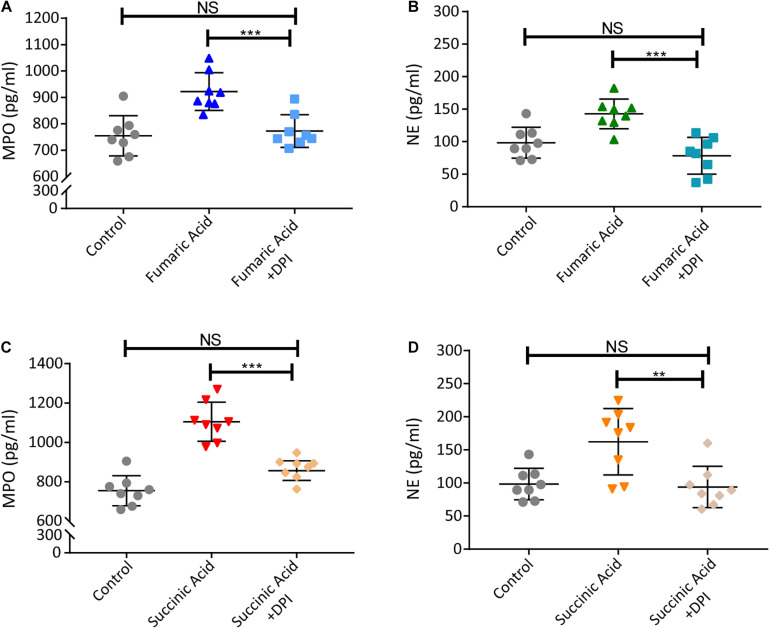
Fumaric acid or succinic acid can induce NET formation via the NADPH oxidase-dependent pathway. **(A,B)** Neutrophils from normal mice were pretreated with DPI followed by the stimulation of fumaric acid. Quantification of MPO and NE in the supernatants of neutrophils. **(C,D)** Neutrophils from normal mice were pretreated with DPI followed by the stimulation of succinic acid. Quantification of MPO and NE in the supernatants of neutrophils (*n* = 8/group; mean ± SD; ***p < 0.001, **p < 0.01; Differences were accessed by one-way ANOVA test).

Previous research has shown that accumulation of fumaric acid or succinic acid can induce activation of NADPH-oxidase and production of ROS (Sudarshan et al., 2009; Chouchani et al., 2014). To investigate whether a similar mechanism was operative in our system, experiments were performed with DPI, a classic inhibitor of NADPH-oxidase, which was used to block neutrophils before fumaric acid or succinic acid stimulation. NET formation was analyzed by quantifying MPO and NE in neutrophil supernatants after stimulation or inhibition. The results showed that DPI was able to inhibit the effect of fumaric acid or succinic acid on the generation of NETs (Figure 6). Taken together, these data confirmed that NET formation could be regulated by fumaric acid and succinic acid via the NADPH oxidase-dependent pathway.

### Fumaric Acid Does Not Activate Th1 and Th17 Cells *in vitro*

Earlier studies have shown that succinic acid does not affect CD4^+^ T cell activation in vitro, although it can promote the proliferation and activation of CD4^+^ T cells in vitro following antigen presentation by dendritic cells (Rubic et al., 2008). We confined our experiments to the effect of fumaric acid on CD4^+^ T cells in vitro, and were not able to show a detectable effect on CD4^+^ T cell differentiation into Th1 or Th17 cells (Supplementary Figure 3).

## Discussion

This study shows that an increase in the housing temperature worsens the development of autoimmune uveitis in mice immunized with a retinal peptide (IRBP). This was associated with an increased frequency of Th1 cells and Th17 cells as well as a higher level of neutrophil activation. Plasma metabolomics showed that under our experimental conditions an increased temperature was shown to increase fumaric acid and succinic acid which were intermediate metabolites of the TCA cycle. Further experiments showed that these metabolites were able to activate neutrophils as evidenced by an increased generation of NETs.

Uveitis can be caused by infectious or non-infectious mechanisms. Non-infectious uveitis is thought to involve a dysregulation of the immune response and can be divided into entities with an autoimmune or autoinflammatory etiology (Yang et al., 2005). Risk factors for the development of uveitis include an interplay between genetic and environmental factors (Zhou et al., 2014; Yuen et al., 2015; Tan et al., 2020). However, little is known concerning the environmental factors. We recently addressed the role of climate change in mainland China and showed that a high ambient temperature is associated with an increased incidence of uveitis (Tan et al., 2020). In the study presented here, we set out to identify the possible mechanisms that might explain the role of ambient temperature on the development of non-infectious uveitis, whereby we used a well-established animal model of autoimmune uveitis (Agarwal et al., 2012).

Several experimental studies have addressed the role of temperature at which animals are housed on their immune response. These studies showed that a high housing temperature could not only affect the growth of animals, but also affected the immune functions and could aggravate inflammation (Tomiyama et al., 2015; Giles et al., 2016; Koch et al., 2019; Moriyama and Ichinohe, 2019). Our data are in agreement with observations from these studies. However, in most research, animal models of heat stress are kept at 30°C, 39°C or even higher temperature. In preliminary experiments we found if the ambient temperature was elevated to 39°C or higher it caused an increased mortality rate of the EAU mice during the experiments (Nonaka et al., 2018; Wang J. et al., 2019). We assume that increased mortality in EAU mice exposed to much higher temperatures might be due to heat exhaustion, heatstroke, or hyperthermia. However, we did not record these phenomena objectively and did not evaluate whether these systemic effects had an effect on the ocular inflammation. Instead, a 30°C environment could reduce the death rate of EAU mice induced by anesthesia. This is why we chose a temperature of 30°C, a relatively mild high temperature environment. In our experiments where EAU mice were treated very shortly at a high temperature (30 degrees), the animals also had obvious inflammation of their eyes, but the manifestation was milder, and nearly the same as seen in mice kept in a normal temperature environment. We assume that a short duration of temperature stimulation has no impact on inflammation and only prolonged high temperature conditions had an effect on the ocular autoimmune response.

Our data are in agreement with previous studies which suggested that the frequencies of CD4^+^ and CD8^+^ T cells could be increased by chronic heat stress (Kokolus et al., 2013; Huo et al., 2019). We also observed that a high ambient temperature could induce higher frequencies of Th1 and Th17 cells, with a concomitant increase of the expression of IFN-γ and IL-17A mRNA -in the spleens and retinas of the EAU.

In addition to helper T cells, macrophages and neutrophils play an important role in autoimmune diseases such as EAU (Merida et al., 2015; Balkarli et al., 2016). According to previous studies on innate immunity and high ambient temperature, macrophages can be activated by heat stress to present autoantigens to T cells and polarize to the so-called M1 subtype (Slawinska et al., 2016; Koch et al., 2019). Little is known, however, on the mechanisms whereby high environmental temperature influences neutrophil function. NET generation, triggered by activated neutrophils, has recently received an increased attention in several autoimmune diseases, such as Behcet’s disease and systemic lupus erythematosus (Leffler et al., 2012; Safi et al., 2018). Consistent with previous studies, a higher NET formation indicates a higher pro-inflammatory status in the affected tissues (Vorobjeva and Pinegin, 2014; Dicker et al., 2018). To our knowledge, we are the first to report an effect of environmental temperature increase on the formation of NETs in EAU. During dissection, we found that the spleens of EAU mice kept at a high ambient temperature were smaller and lighter. However, according to former research on inflammation, animals with severer inflammation would have larger spleens. We suppose that the high ambient temperature may affect the spleen volume of EAU mice via hormonal alterations, but there is not enough research supporting this speculation at present (Leceta and Zapata, 1985) and this hypothesis awaits further investigation.

Previous studies have suggested that the intermediates α-ketoglutaric acid, fumaric acid and succinic acid of the aerobic respiration-related TCA cycle were increased in plasma from animals kept at a high ambient temperature (Shi and Wang, 2016; Nonaka et al., 2018). Succinic acid can induce higher frequencies of helper T cells by amplifying the antigen presentation of monocytes through succinate receptor 1, but failed to activate T cells by itself (Rubic et al., 2008; Saraiva et al., 2018). These data are consistent with the results of the animal or cell studies we present here. Interestingly, fumaric acid or succinic acid accumulation may also enhance the TCA cycle, accelerating ROS production (Sudarshan et al., 2009; Ren et al., 2014). Previous studies indicate that the presence of ROS (NADPH oxidase-dependent) contributes to M1 polarization in macrophages and is also an important activator of NET formation (Branzk and Papayannopoulos, 2013; Chouchani et al., 2014; Vorobjeva and Pinegin, 2014; Dabrowska et al., 2016; Ko et al., 2018; Rendra et al., 2019). Our data showed that both fumaric acid and succinic acid can significantly increase the formation of NETs. Meanwhile, DPI could successfully inhibit fumaric acid or succinic acid from inducing NET formation. Therefore, we confirmed that increased NET formation induced by fumaric acid or succinic acid was NADPH oxidase-dependent. These data suggest a possible causality between a high temperature and NET production, although further studies are needed to confirm these observations and to exclude other possible mechanisms of action.

Our study has several limitations. First, to observe possible effects on intraocular inflammation more clearly, we used a mild EAU model in this study, which is different from our earlier research on EAU mice (Huang et al., 2018, 2020). Second, due to a limited availability of experimental equipment, we only investigated the EAU development at only two levels of ambient temperature and did not measure the influence on the immune system of EAU mice prior to or after immunization at more time periods of heat stress. Since the majority of previous studies on EAU used 6–8 weeks old female mice or rats, we used a similar approach, but further study is needed to investigate whether our conclusions can be extrapolated to older mice and whether the same findings will also be seen in male animals (Agarwal et al., 2012). Third, the MPO staining in retinal tissues was too light to reliably quantify the differences. More sensitive techniques are necessary to address this issue. Finally, we assumed that energy metabolism was probably the most relevant target influenced by temperature, but other pathways or systemic effects may of course also be affected by temperature changes. A further limitation is that among the 17 differentially expressed metabolites detected, we only concentrated on the role of fumaric acid, succinic acid and NADPH oxidase, but other mechanisms cannot be excluded and deserve further study.

In conclusion, this study indicates that an increased ambient temperature is a risk factor for the development of uveitis. An increase in the ambient temperature was shown to upregulate the frequencies of Th1 and Th17 cells as well as the formation of NETs. The effect on neutrophils was probably mediated via the NADPH oxidase-dependent pathway. This study is the first to illustrate that a high ambient temperature has an effect on an autoimmune model of ocular inflammation and these findings may provide potential targets for the design of anti-inflammatory therapies and will hopefully promote policies to mitigate the burden of disease caused by global warming.

## Data Availability Statement

The raw data supporting the conclusions of this article will be made available by the authors, without undue reservation.

## Ethics Statement

The animal study was reviewed and approved by the Animal Care and Use Committee of The First Affiliated Hospital of Chongqing Medical University.

## Author Contributions

PY and SP conceived the idea and designed the experiments. SP and HT performed all the experiments. SP, HT, and RC analyzed the data. QW contributed to FCM test and immunofluorescence. YZ, LC, and HL contributed to animal modeling. SP and HT wrote the manuscript. CZ and QC contributed to experimental equipment. PY, GS, and AK interpreted the data and revised the manuscript. All authors contributed to the article and approved the submitted version.

## Conflict of Interest

The authors declare that the research was conducted in the absence of any commercial or financial relationships that could be construed as a potential conflict of interest.
