# Supplementary material for: High Ambient Temperature Aggravates Experimental Autoimmune Uveitis Symptoms
Source: Front Cell Dev Biol. 2021 Mar 25;9:629306. doi: 10.3389/fcell.2021.629306 (PMC8027130; doi:10.3389/fcell.2021.629306)
Supplement: Supplementary file 1 [file Data_Sheet_1.pdf]

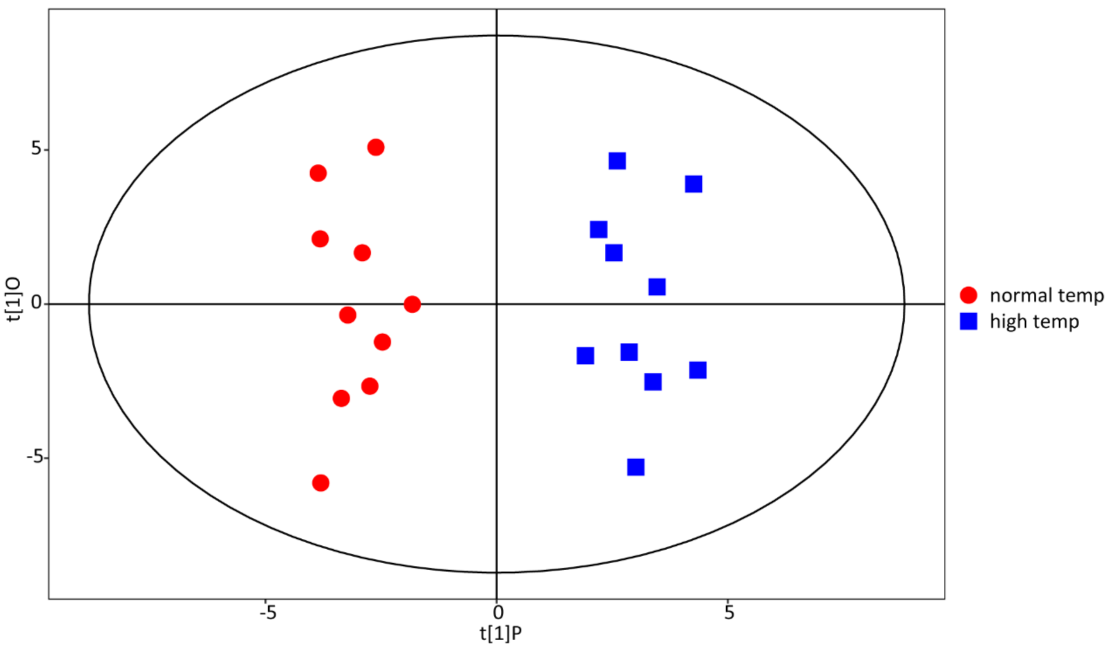

Figure S1. Alteration of metabolites between the EAU mice at normal and high temperature. OPLS-DA of fecal samples from the mice at high temperature (blue square) and normal temperature (red circle). (n = 10/group).

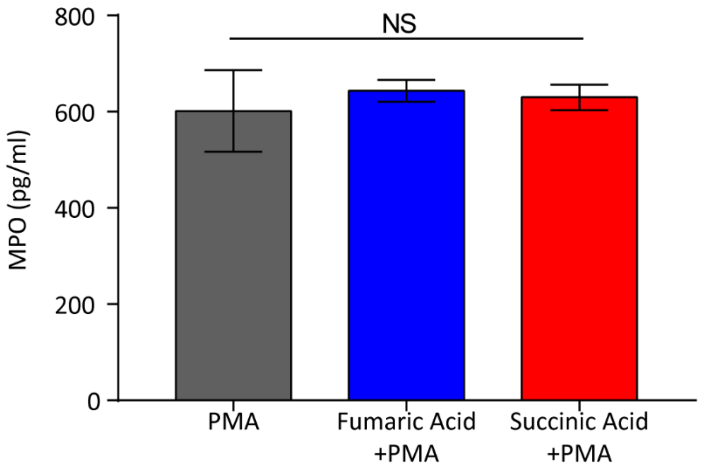

Figure S2. PMA-induced NET formation of neutrophils pre-treated with fumaric acid or succinic acid. Quantification of MPO from normal mice treated with fumaric acid or succinic acid. (n_{PMA} = 12, $n_{Fumaric\ Acid}$ = 6, $n_{Succinic\ Acid}$ = 6; mean ± SD; No statistical differences; Differences were accessed by one-way ANOVA test).

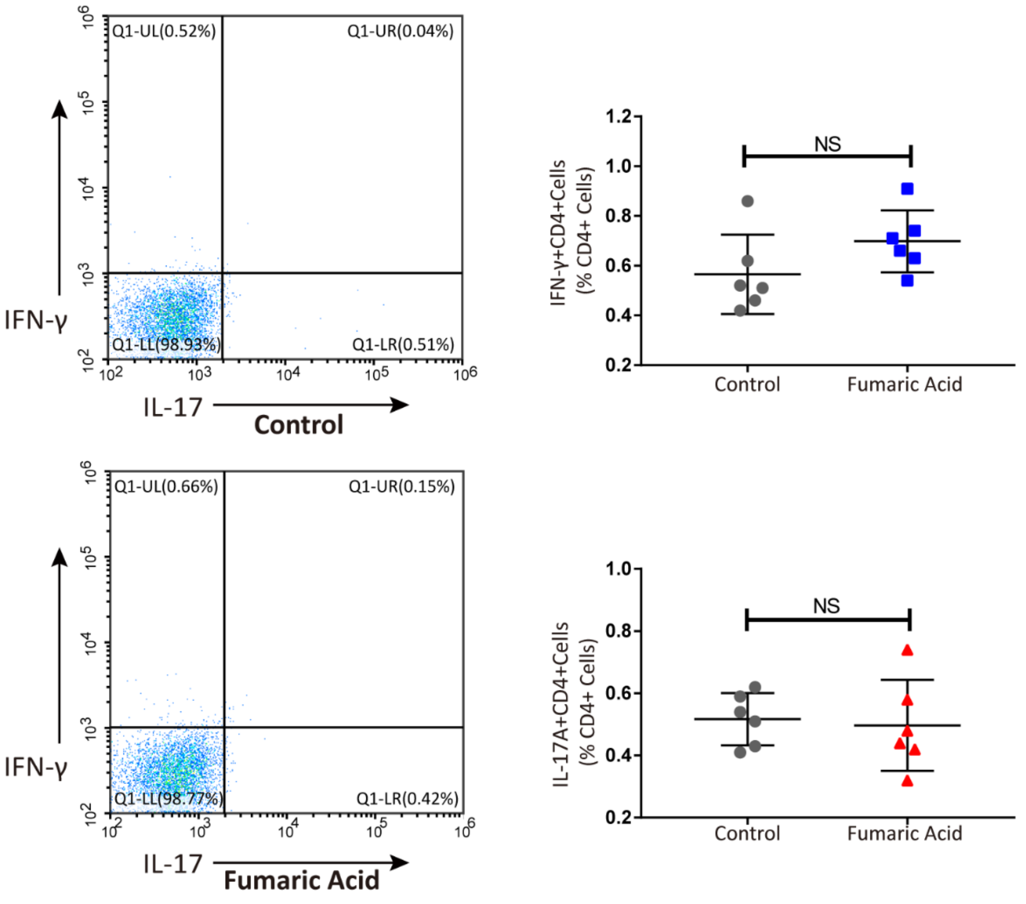

Figure S3. Fumaric acid had no effect on CD4+ T cells in vitro. Representative and quantification of IFN-γ+CD4+ T cells and IL-17A+CD4+ T cells from normal mice treated with fumaric acid or DMSO as control. (n = 6/group; mean ± SD; No statistical differences; Differences were accessed by Unpaired t test).
